# Comparative Mitogenomics of Four *Anomala* Species and a Phylogenetic Reassessment of Scarabaeidae

**DOI:** 10.3390/genes17080949

**Published:** 2026-08-13

**Authors:** Guang Zeng, Jing Yin, Jianrong Huang, Xianyi Wang, Songxian Yan

**Affiliations:** 1 Department of Resources and Environment, Moutai Institute, Renhuai 564500, China; zengguang1992@126.com (G.Z.); 15937715938@163.com (J.Y.); huangjianrong0702@163.com (J.H.); 2 Department of Education of Guizhou Province, Engineering Research Center of Health Medicine Biotechnology, Guiyang 561113, China

**Keywords:** insect, Scarabaeidae, phylogenetic analysis, mitogenome

## Abstract

**Background**: The genus *Anomala* is exceptionally species-rich and includes many species recognized as major agricultural and forest pests. Nevertheless, the taxonomic status of certain species remains contentious, and the phylogenetic relationships within the genus are still poorly resolved and in need of further investigation. **Objectives**: To better elucidate the evolutionary relationships among these insects, we assembled the complete mitochondrial genomes (mitogenomes) of four *Anomala* species—*A. aulax*, *Anomala* sp., *A. vitalisi*, and *A. xantholoma*. Multiple molecular datasets were integrated to reconstruct the phylogenetic framework of Scarabaeidae. Methods: Wild adult specimens of four *Anomala* species were collected. After total DNA extraction, complete mitogenomes were assembled and annotated for comparative analysis of core genomic traits. Multiple datasets were applied to construct phylogenetic trees via maximum likelihood and Bayesian inference, so as to evaluate the evolutionary characteristics of each taxon. **Results**: Our analyses reveal that the mitogenomes of *Anomala* species exhibit a conserved architecture, characterized by similarities in genome size, AT nucleotide bias, and gene order, suggesting a high degree of evolutionary stability within the genus. The resulting phylogenetic trees robustly support the monophyly of the following families: Passalidae, Lucanidae, Geotrupidae, Trogidae, Hybosoridae, and Glaphyridae. Additionally, the phylogenetic findings revealed the paraphyly of both Scarabaeidae and *Anomala*, and further suggested that Melolonthinae is likely a paraphyletic group. **Conclusions**: This study enriches the currently available mitogenomic data and provides a preliminary foundation for understanding the phylogeny, resource conservation, and genetic diversity of *Anomala*.

## 1. Introduction

The family Scarabaeidae (Coleoptera: Polyphaga: Scarabaeoidea) is exceptionally species-rich [1]. As major agricultural and forestry pests, scarab beetles cause severe damage through both larval and adult feeding. The larvae, commonly known as grubs, attack the roots of crops and trees, often leading to rapid plant decline or death and resulting in substantial productivity losses. Adults, in turn, consume the foliage of a wide range of plants, including seedlings, garden flowers, seeds, and rhizomes of crops, lawns, and pastures. Their intense herbivory can quickly devastate extensive vegetation or entire forests, compromising tree health and stand quality. Moreover, the diverse morphological traits and burrowing behaviors exhibited across species pose considerable challenges for pest management [2,3]. Accurate species identification is therefore essential for implementing targeted and cost-effective control strategies [4]. However, the phylogenetic classification of Scarabaeidae remains complicated. In particular, classification systems based on morphological features—such as abdominal valves, genitalia, palates, and prosternal processes—have undergone continual revision [5,6,7].

Scarabaeidae, as a taxonomically significant group, has long been plagued by controversies surrounding the monophyly and higher-level placement of its constituent subfamilies [1]. Both morphological and molecular evidence support a close relationship between Rutelinae and Dynastinae, often recovered as a clade; however, the respective monophylies of these two subfamilies remain to be rigorously tested. Three main hypotheses have been proposed regarding their phylogenetic affinity: that Dynastinae represents the ancestral lineage of Rutelinae, that the two subfamilies are sister groups, or that their relationship remains unresolved [5]. In addition, the taxonomic status of the tribe Adoretini is contentious. Originally described within Melolonthinae, it was later transferred to Rutelinae, yet this placement has not gained unanimous acceptance. Collectively, these issues highlight that the evolutionary boundaries and circumscription of Rutelinae remain far from a scholarly consensus [8,9]. The taxonomy of many genera within this family has been subject to ongoing revision [10]. Morón (1983), in his revision and description of certain species, employed male external genitalia characters for taxonomic studies at the tribal and subgeneric levels. García-Vidal (1984), in describing 11 species of the genus *Phyllophaga* Harris, similarly utilized male genital morphology, while further noting that many structures of the female external genitalia are of limited diagnostic value for species identification.

The genus *Anomala* represents the taxon with the most concentrated taxonomic difficulties within the group. With over 1000 species recorded globally, this genus has yet to be subdivided into subgenera. Its constituent species exhibit remarkable similarities in external morphology, making the discrimination of closely related taxa extremely challenging. In addition, the genus harbors a substantial number of historical problems, including numerous synonyms and misplacements in taxonomic assignment [11]. Molecular evidence, however, has revealed new complications. A phylogenetic analysis of Rutelinae indicated that the generic assignments within the tribe Anomalini require re-evaluation. For instance, the analysis showed that Anisoplia is rendered paraphyletic by being “broken” by an internal clade containing Hemichaeioplia, Brancoplia, and Chaetopteroplia. This suggests that the higher-level classification system within Anomalini may be broadly plagued by non-natural circumscriptions [12]. In addition, Scarabaeidae are holometabolous insects, exhibiting extensive morphological differences between larvae and adults. If species identification relies solely on the morphological characteristics of adults, misidentification may occur—even by experienced entomologists. Furthermore, some morphological features of insects may be influenced by environmental factors, thereby compromising the accuracy of species identification based on such traits.

As an independent genetic system outside the nucleus, the mitochondrial genome (mitogenome) is characterized by rapid evolution, lack of recombination, high copy numbers, and maternal inheritance [8,9,10,11,12,13]. With the development of next-generation sequencing (NGS) technology, mitogenomes have been widely applied in molecular phylogenetics, population genetics, molecular diagnostics, and phylogeography in entomology [9,10,11,12,13,14]. Mitogenomic analysis has become a well-established and effective adjunct to traditional taxonomic research [10,15,16]. However, the mitogenomic database for Scarabaeidae remains insufficient, and additional molecular data are urgently needed to fill this knowledge gap [12,17,18,19,20,21].

In this study, we sequenced the mitogenomes of four *Anomala* species (*A. aulax*, *Anomala* sp., *A. vitalisi*, and *A. xantholoma*) with three main objectives: characterizing their mitogenomic composition (base composition, evolutionary rates, and conserved sequences), reconstructing the phylogeny of Scarabaeidae, and evaluating the monophyly of its constituent subfamilies.

## 2. Materials and Methods

### 2.1. Sample Collection, DNA Isolation, and Sequencing

Samples were collected by light-trapping at night or net-trapping during the day, and were preserved either by drying or in absolute ethanol. Specimens of the four *Anomala* species were collected from Guizhou, Sichuan, and Yunnan Provinces, China (detailed collection information is provided in Table S1). Species identification was primarily based on male genitalia, supplemented by other morphological characters, with taxonomic terminology following Bouchard (2011). To maximize the representation of distinct morphological traits, we included *Anomala* sp. in this study, as it exhibits substantial morphological divergence from the other species examined. Following examination and identification, the dried specimens were deposited at Guizhou Medical University. Each specimen was initially identified based on morphological features and subsequently confirmed by molecular analysis. Genomic DNA was extracted from the thoracic muscle of adult males using the DNeasy Blood & Tissue Kit (Qiagen, Germantown, MD, USA) according to the manufacturer’s instructions. The DNA samples were stored at −20 °C until sequencing. Sequencing was performed on the Illumina HiSeq 4000 platform, generating approximately 4 Gb of raw data per sample (Berry Genomics, Beijing, China) [22].

### 2.2. Assembly, Genome Annotation, and Sequence Analysis

The raw reads were assembled using Geneious version R9 (https://www.geneious.com), and the resulting sequences were compared with the homologous sequence of *A. russiventris* (NC065310). Species identification was further confirmed via BLAST version 2.16.0 searches against the NCBI database, using a threshold of >99% sequence similarity for the *cox1* gene. The mitogenomes of the four *Anomala* species were annotated using the MITOS server on the Galaxy platform [23] and genetic code Table S5. The locations of the ribosomal large subunit (rrnl) and ribosomal small subunit (rrns) were determined via comparison with homologous mitogenomes. The locations of 13 protein-coding genes (PCGs) were confirmed using ORF Finder via Geneious. Finally, all genes were compared with the reference to verify their validity. Four circular mitogenomic maps were constructed using OGDraw version 1.3.1 [24]. Nonsynonymous substitution rates (Ka) and synonymous substitution rates (Ks) of 13 PCGs (*atp6*, *atp8*, *cox1*, *cox2*, *cox3*, *cob*, *nad1*, *nad2*, *nad3*, *nad4*, *nad4l*, *nad5*, and *nad6*) in *Anomala* mitogenomes were calculated using DnaSP version 6.12.03 to elucidate the genomic evolution of *Anomala* [25]. Gene synteny analysis of the mitogenomes of the four *Anomala* species was performed using Mauve version 2.4.0 via Geneious [26]. The data that support the findings of this study will be available from GenBank at https://www.ncbi.nlm.nih.gov/ (access on 10 August 2025), with accession number PQ139503-PQ139506 (*Anomala aulax* PQ139506, *Anomala* sp. PQ139505, *A. vitalisi* PQ139504, and *A. xantholoma* PQ139503).

### 2.3. Phylogenetic Analysis

To explore the phylogenetic relationships of the genus *Anomala*, we reconstructed a phylogenetic tree of Scarabaeidae based on the mitogenomes of 73 species. Complete mitogenomes of Elateriformia and Cucujiformia species were also included [as outgroups] to root the tree. *Abscondita anceyi* and *Aclees cribratus* were used as outgroups (Table S2) [27,28].

Phylogenetic relationships within Scarabaeidae were reconstructed using the 13 protein-coding genes (PCGs) mentioned above. Each PCG sequence was aligned using the MAFFT algorithm via the Translator X platform and the MAFFT version 7.0 online server with the G-INS-i strategy [29,30]. Subsequently, the resulting 13 alignments were assessed and manually corrected using MEGA 7.0 [31]. Three datasets were generated for phylogenetic inference: the 13PCG dataset (all codon positions of the 13 PCGs), the PCG12 dataset (first and second codon positions only), and the AA dataset (amino acid sequences of the 13 PCGs).

Phylogenetic trees were reconstructed using both maximum likelihood (ML) and Bayesian inference (BI) methods. ML analysis was performed with IQ-TREE version 1.6.12, with node support assessed via 1000 ultrafast bootstrap replicates [32]. BI analysis was conducted using MrBayes version 3.2.7 under default settings, with four independent runs of 100 million generations each, sampled every 1000 generations [33]. Convergence was considered acceptable when the average standard deviation of split frequencies fell below 0.01, at which point the first 25% of sampled trees were discarded as burn-in. The remaining trees were used to generate a consensus tree and to calculate posterior probabilities. Phylogenetic trees were visualized using FigTree version 1.4.4 (tree.bio.ed.ac.uk/software/figtree/, access on 11 August 2025) and refined for presentation using Adobe Illustrator CC version 22.1.

## 3. Results

### 3.1. General Features of the Four Anomala Mitogenomes

**Figure 1:**
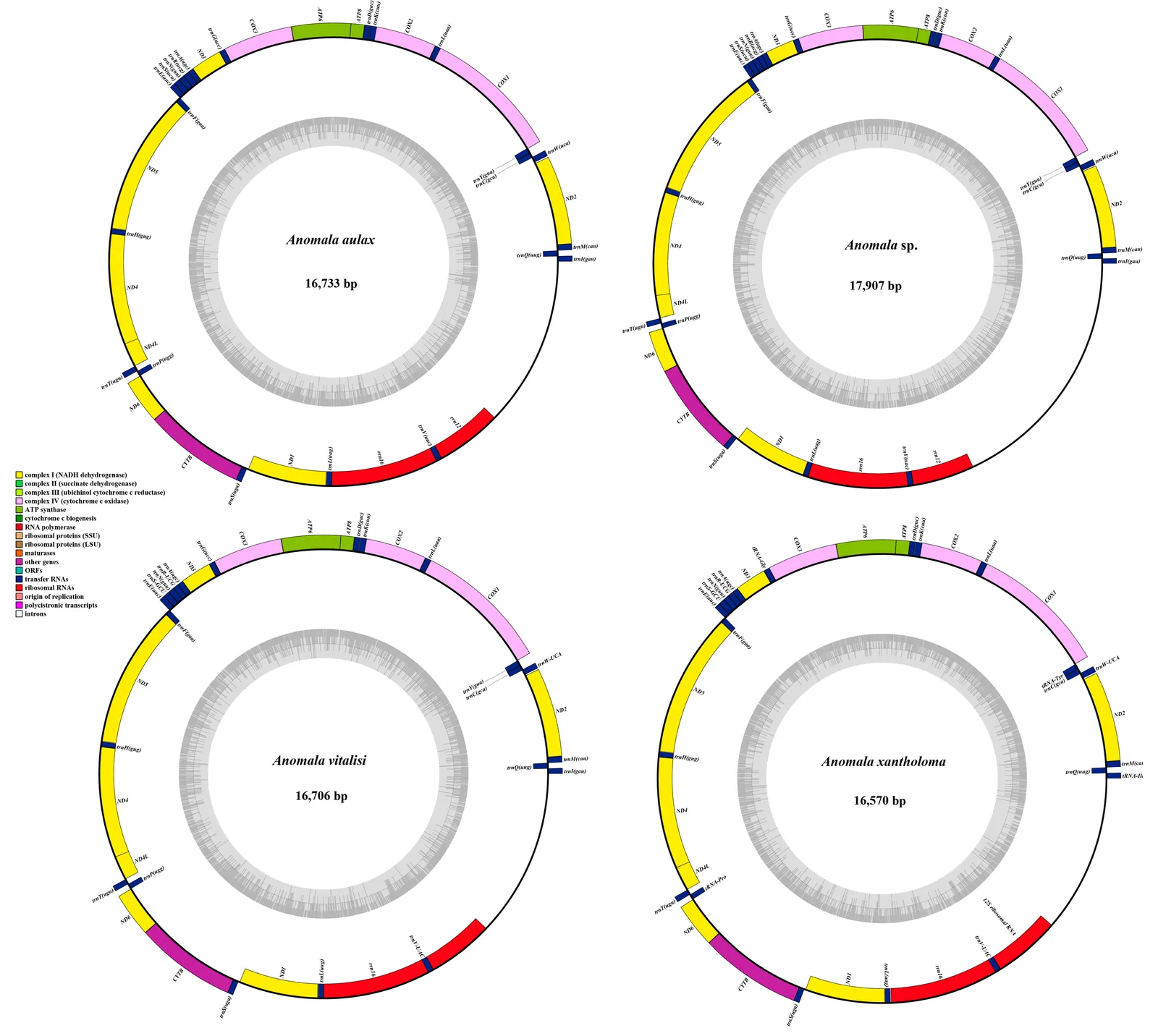
Circular maps of four *Anomala* mitogenomes. Genes are indicated by different colored blocks.

The complete mitogenomes of the four *Anomala* species are circular DNA molecules (Figure 1), each containing the canonical set of 37 genes: 13 protein-coding genes (PCGs), 22 transfer RNA (tRNA) genes, and 2 ribosomal RNA (rRNA) genes. The lengths of the sequenced mitogenomes were 16,733 bp (*A. aulax*), 16,570 bp (*A. xantholoma*), 17,907 bp (*Anomala* sp.), and 16,706 bp (*A. vitalisi*). The comparison of the obtained mitogenomes with those in the NCBI database revealed that *A. russiventris* (15,601 bp) had the shortest mitogenome length, whereas *Anomala* sp. (17,907 bp) had the longest. The longest and shortest in all four *Anomala* mitogenomes were all 1716 bp long (nad5). The length of atp8 was the shortest (156 bp) in all four *Anomala* mitogenomes. These two PCGs are highly conserved in all insect species, especially atp8, which is highly conserved at 150–156 bp.

**Figure 2:**
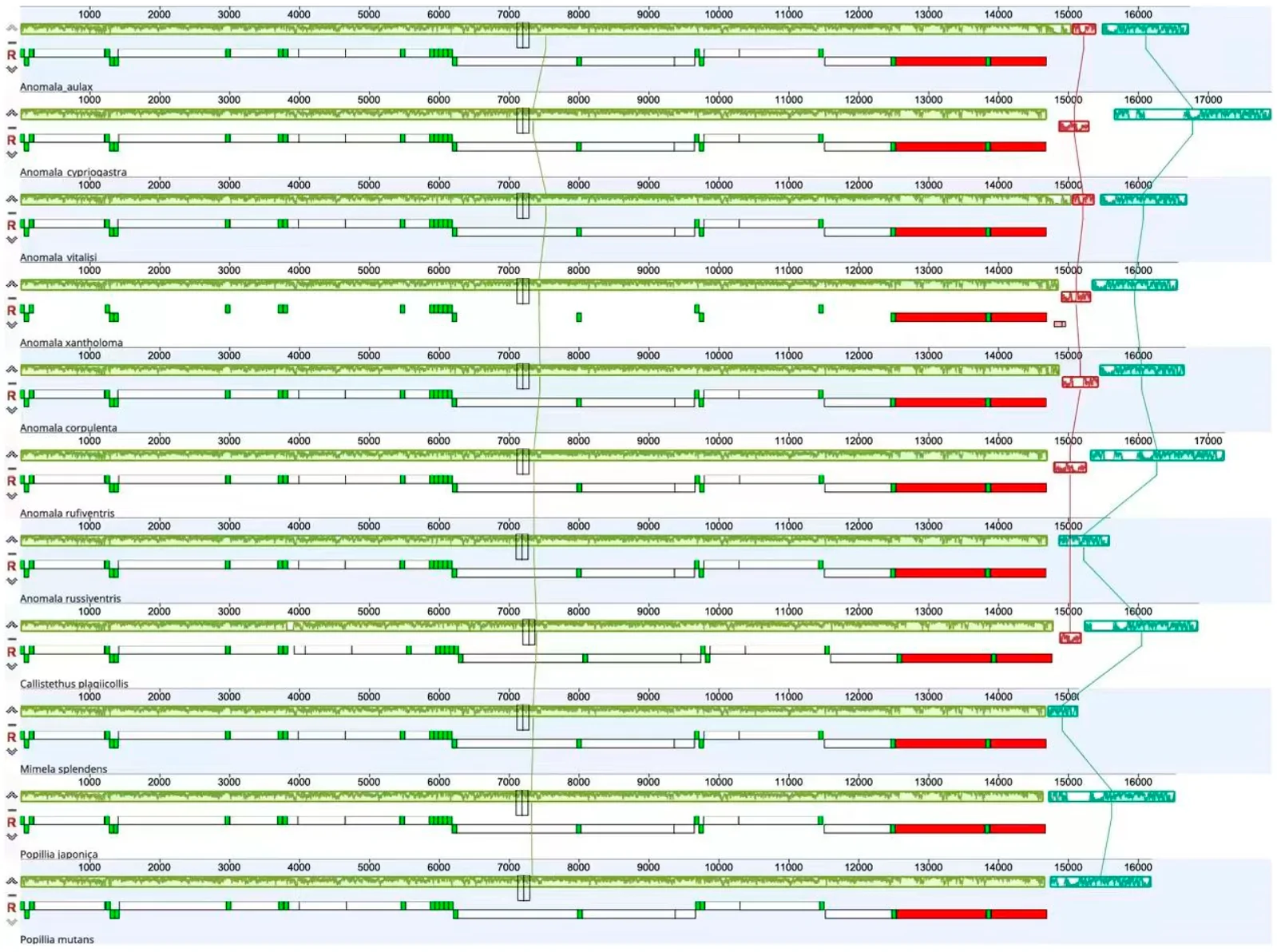
Collinearity analysis map of 11 Rutelinae mitogenomes, showing three homologous regions. The sizes and positions of the homologous regions varied across the mitogenomes.

The mitogenome of *Anomala* is extremely conservative, and its sequence is as follows: *trnI*-*trnQ*-*trnM*-*nad2*-*trnW*-*trnC*-*trnY-cox1*-*trnL1*-*cox2*-*trnK*-*trnD*-*atp8*-*atp6*-*cox3*-*trnG*-*nad3-trnA-trnR*-*trnN*-*trnS1*-*trnE*-*trnF*-*nad5*-*trnH*-*nad4*-*nad4l*-*trnT*-*nad6*-*cytb*-*trnS2*-*nad1*-*trnL2*-*rrnL*-*trnV*-*rrnS*. Most genes were tightly arranged with no substantial overlaps or gaps. Comparative mitogenomic analysis of *Anomala* revealed a highly conserved 16-bp fragment located between *trnS* and *nad1*. Collinearity analysis further identified three conserved regions within the Rutelinae mitogenome: the first lies within the *nad5* gene, while the second and third are situated in the control region. Notably, the first and third conserved regions were present across all examined species (Figure 2).

The structural characteristics of the mitochondrial genomes of the four *Anomala* species in this study are highly consistent with those previously reported for Scarabaeidae species: all are circular DNA molecules containing 37 standard genes, including 13 PCGs, 22 tRNAs, and 2 rRNAs.

### 3.2. Evolutionary Rates of Scarabaeidae

**Figure 3:**
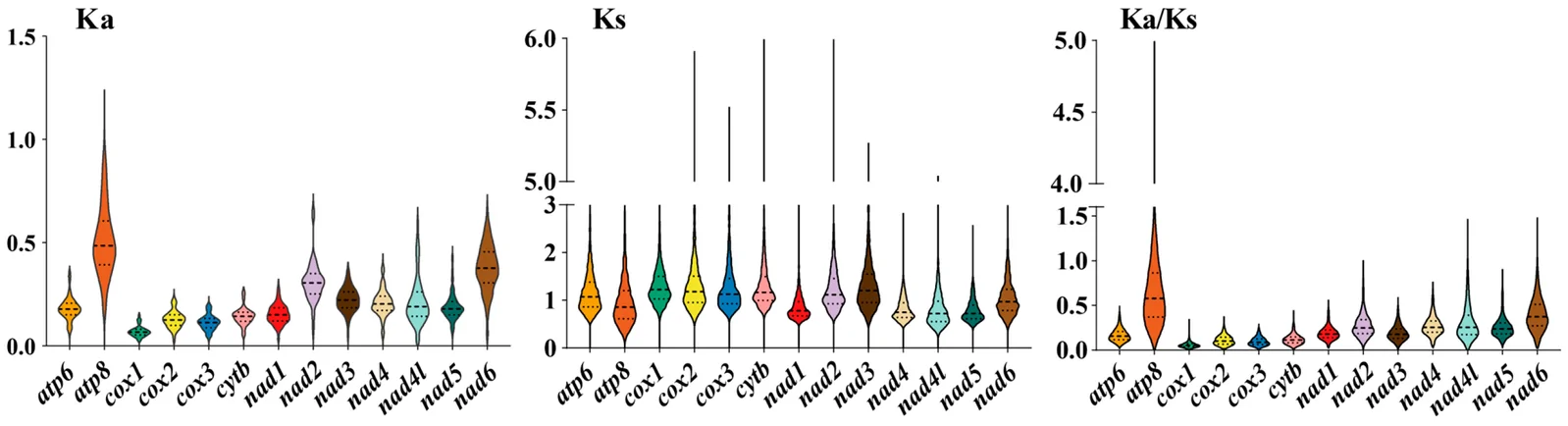
Genetic analysis of 13 protein-coding genes in 4 *Anomala* species. K2P, Kimura-2-parameter distance; Ka, number of nonsynonymous substitutions per nonsynonymous site; Ks, number of synonymous substitutions per synonymous site.

To further elucidate the evolutionary dynamics of Scarabaeidae, we analyzed the protein-coding genes (PCGs) for selection pressure at the amino acid level. The nonsynonymous substitution rate (Ka) of *atp8* was significantly higher than that of the other PCGs, whereas *cox1* exhibited the lowest Ka among the 13 genes. Overall, the Ka values of all 13 PCGs were below 0.5. Among these genes, *cox2*, *cytb*, and *nad2* showed the highest synonymous substitution rates (Ks), while nad4 had the lowest, with all Ks values falling below 0.8. These results indicate that purifying selection predominates at the amino acid level across all species. Furthermore, *cox1* and *cox3* exhibited significantly lower Ka/Ks ratios, suggesting that these genes have experienced stronger evolutionary constraints (Figure 3).

### 3.3. Phylogenetic Relationships of Scarabaeidae

**Figure 4:**
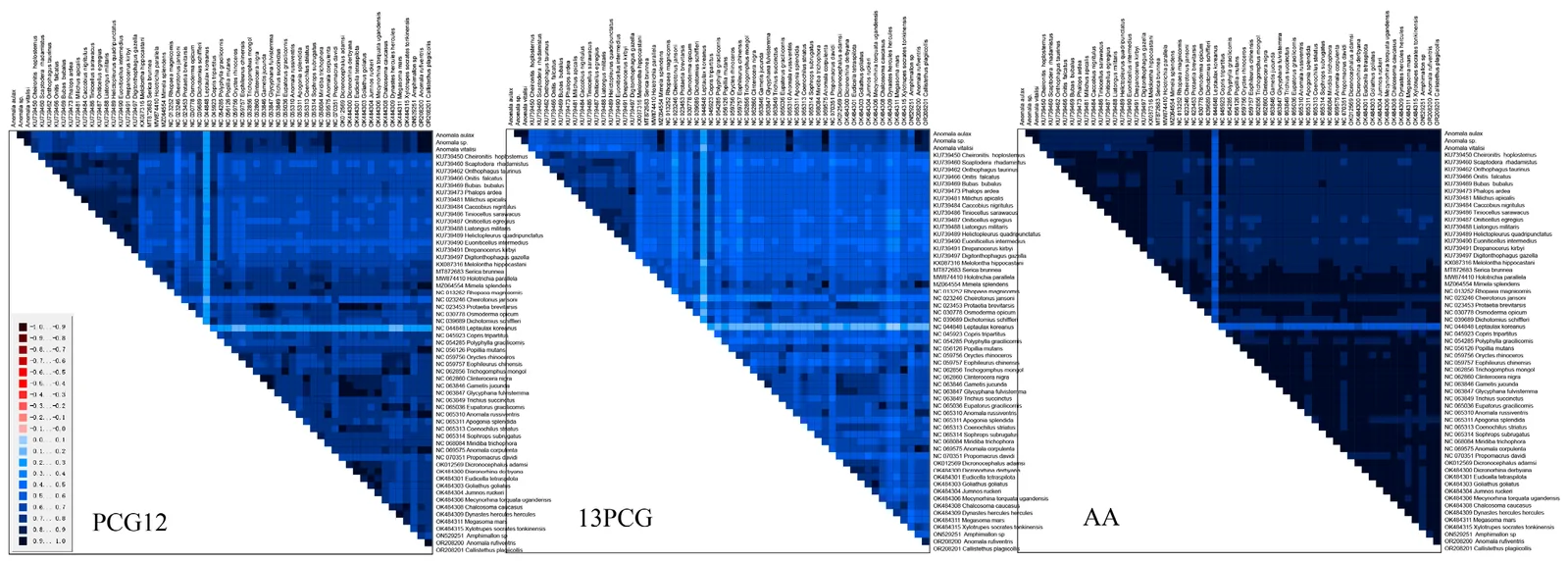
Heterogeneity in PCG12, 13PCG, and AA datasets of the mitogenome of Scarabaeidae. Differences in heterogeneity between the sequences are indicated by color, with dark red (−1) to dark blue (+1) representing differences from heavy to light, respectively.

**Figure 5:**
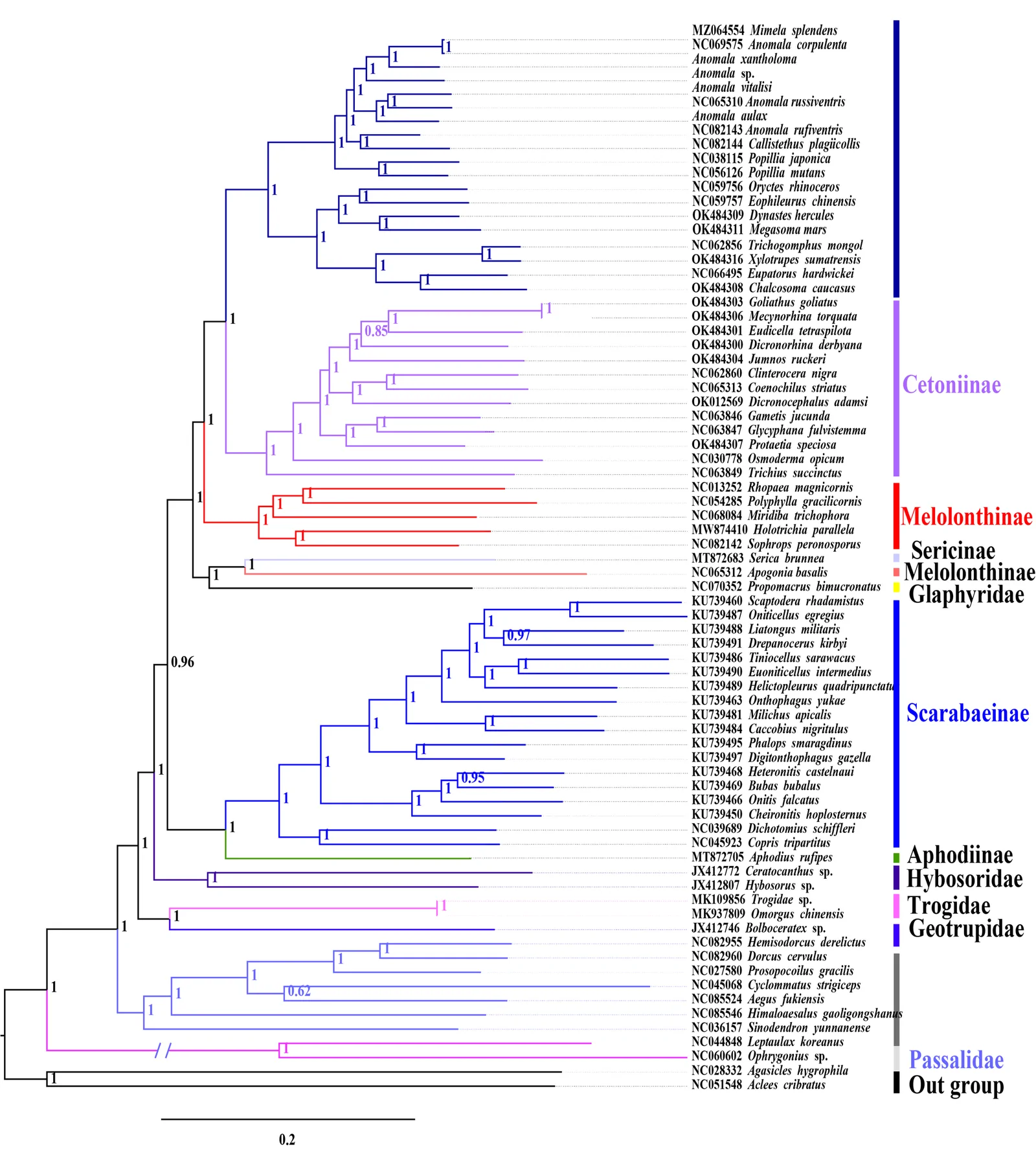
Molecular phylogeny of Scarabaeidae based on Bayesian inference analysis of 13 protein-coding genes using MrBayes 3.2.7.

We used AliGROOVE version 1.07 software to detect heterogeneity in the three datasets (AA, PCG12, and 13PCG) and to evaluate their suitability for phylogenetic reconstruction (Figure 4). Heterogeneity analysis revealed that the 13PCG dataset exhibited the highest heterogeneity, while the AA dataset showed the lowest. Notably, the heterogeneity scores for all datasets were <0.1, indicating low overall heterogeneity and thus sufficient reliability for phylogenetic inference. Consequently, all three datasets were considered suitable for tree construction. Maximum likelihood (ML) and Bayesian inference (BI) analyses of the three datasets yielded almost identical topologies across all six inferred trees (Figure 5 and Figures S1–S5). The BI trees exhibited consistently high support, with posterior probabilities (PP) ranging from 0.95 to 1.00; only two nodes had support values below 0.95. In comparison, the support values from ML analysis were notably lower than those from BI analysis. Among the three datasets, the 13PCG dataset produced the highest nodal support, likely due to its greater informational content.

At the family level, our phylogenetic results supported the monophyly of Passalidae, Lucanidae, Geotrupidae, Trogidae, Hybosoridae, and Glaphyridae. The monophyly of Scarabaeidae was also well supported, with its subfamilies Melolonthinae (Apogonia splendida) and Sericinae (Serica brunnea) nested within the Scarabaeidae clade. Notably, Glaphyridae (Propomacrus bimucronatus), an independent family within Scarabaeoidea, formed a sister lineage to Scarabaeidae, a placement consistent with the classical taxonomic framework.

Within Rutelinae, the monophyly of three genera—*Callistethus*, *Mimela*, and *Popillia*—was well supported, consistent with the evolutionary relationships of closely related genera in this subfamily. In contrast, *Anomala*, the focal genus of this study, was recovered as non-monophyletic, with its species scattered across multiple clades interspersed with *Callistethus* and *Mimela*. This paraphyletic pattern is congruent with the widely recognized complex evolutionary history of *Anomala*.

## 4. Discussion

In this study, we analyzed and characterized the mitogenomes of four scarab beetle species and identified a conserved intergenic fragment between *trnS* and nad1 in the Scarabaeidae mitogenome. This fragment, ranging from 16 to 31 bp in length, has been reported to be common among scarabaeoid families [18,34]. In the *Anomala* mitogenomes examined here, this interval was 16 bp, representing a relatively conserved region [35,36]. This fragment may therefore serve as a robust marker for future molecular identification efforts. Among the 13 PCGs analyzed, *atp8* exhibited significantly higher Ka/Ks values. With the exception of atp8, all other genes had Ka/Ks ratios between 0 and 1, indicating that they are predominantly under negative (purifying) selection. In contrast, cox1 displayed the lowest Ka/Ks value, suggesting that it experiences the strongest purifying selection pressure. This result supports the widely recognized utility of *cox1* as an ideal DNA barcode marker.

This genomic composition fully conforms to the typical architecture of scarabaeid mitochondrial genomes, as observed in the four dung beetle species (Scarabaeinae) reported by Zhang et al. and the two phytophagous scarab species (Cetoniinae, Melolonthinae) sequenced by Guo et al. [3,21].

Regarding gene rearrangement, no gene rearrangements were detected in the mitochondrial genomes of the *Anomala* species in this study, and the gene order is highly conserved. In contrast, Zhang et al. identified two novel rearrangements in coprophagous dung beetles: a reciprocal translocation of *trnT*/*trnP* in *C. magicus* and a long-distance translocation of *trnI* in *L. bucerus* [3]. Meanwhile, the phytophagous scarabs studied by Guo et al. exhibited no gene rearrangements [21]. These findings indicate that the incidence of mitochondrial gene rearrangements is substantially lower in the phytophagous subfamily Rutelinae than in the coprophagous subfamily Scarabaeinae, and that the genomic structure of *Anomala* is evolutionarily more conserved.

Furthermore, a highly conserved 16 bp fragment was identified between *trnS* and *nad1* in this study, providing a novel stable molecular marker for Scarabaeidae and supplementing the conserved region features not reported by Zhang et al. or Guo et al. [3,21]. Additionally, the AT base bias observed in the mitochondrial genomes of the *Anomala* species in this study is entirely consistent with the base composition preferences of scarabaeid species reported in both reference studies, representing a shared core characteristic of scarabaeid mitochondrial genomes.

In all phylogenetic trees constructed via BI and ML analyses using three different datasets (PCG12, 13PCG, and AA), some species were clustered in pairs with extremely short branches of evolution. We performed comparative mitogenomic analysis to verify the relationships among these clustered species. By comparing the two mitogenomes, we found that except for the control region sequence, the other gene sequences were highly similar (>99%). To verify the attribution of these two species, we first downloaded the cox1 sequence of *Goliathus* from NCBI (*Goliathus goliatus* JX234183; *G. orientalis* JX234186). When the two sequences were compared with the *cox1* sequences of *G. goliatus* OK484303 and *Mecynorhina torquata* OK484306 [37], the similarity was 85.2%. In contrast, *G. goliatus* JX234183-*cox1* and *G. orientalis* JX234186-*cox1* were only 82.5% similar to *G. goliatus* JX234183 (*cox1*) and *G. goliatus* OK484303 (mitogenome)-*cox1*. Therefore, the identification of *G. goliatus* OK484303 was erroneous. Subsequently, we retrieved the *cox1* sequence of another genus, *Mecynorhina* (*M. polyphemus* JX234187 and *M. torquata* JX234186), from NCBI. We aligned these two sequences with the *cox1* sequences of *G. goliatus* OK484303 and *M. torquata* OK484306 and found 90% similarity. Based on our findings, we inferred that both *G. goliatus* and *M. torquata* were putatively misidentified. They were not *Goliathus* species but were more likely to be *Mecynorhina* or a related species. Using the same method, we showed that *Omorgus chinensis* was also misidentified. Similarly, in Rutelinae, *A. corpulenta* and *M. splendens*, which are clustered in all phylogenetic trees and have extremely short evolutionary branches, have up to 99.2% similarity. Furthermore, the results of the *cox1* sequence alignment of these two species showed that *M. splendens* is an *Anomala* species, which confirms the misidentification of *M. splendens*. The mitogenome is a robust tool for accurate identification of species and determination of phylogenetic relationships among insect lineages.

Phylogenetic analysis using the three datasets revealed that *Propomacrus* (Glaphyridae) was clustered with Melolonthinae and Sericinae (Scarabaeidae). Furthermore, *Apogonia* was separated from Melolonthinae and formed a sister group with *Serica* (Sericinae), with a high support rate. This study supports that Cetoniinae, Dynastinae, Rutelinae, Scarabaeinae, and Sericinae are monophyletic groups, while Melolonthinae is non-monophyletic, which is entirely consistent with the mitochondrial phylogenetic conclusions of Guo et al. [21]. Currently, the general presumption is that fecal-eating insects evolved before herbivorous insects [38]. Some researchers believe that the Scarabaeidae is the most primitive family [39], whereas others believe that the stag beetle family Lucanidae is the most primitive family [40].

The phylogenetic relationships obtained in this study support facts related to the feeding habits of the golden turtle, i.e., the fecal-feeding Passalidae are located at the base of the phylogenetic tree, while the wood-feeding Lucanidae are located in the middle of the phylogenetic tree. Furthermore, Cetoniinae and Rutelinae are located at the top of the phylogenetic tree. The results of our phylogenetic analysis support the view that the feeding patterns of scarab insects are closely related to their evolution.

Our study shows that *Anomala* is divided into three groups by *Callistethus*, *Mimela* and *Popillia*. Clade I consists of *A. rufiventris* and *Callistethus plagiicollis* combined, while Clade II is formed by *A. russiventris* and *A. vitalisi*. While *A. aulax* was clustered into one branch, Clade III is composed of *Anomala* sp., *A. xantholoma*, *A. corpulenta*, and *Mimela* splendens. We believe that *Anomala* needs further revision; however, due to the lack of data (especially the two genera of *Mimela* and *Callistethus*), it is not possible to carry out the revision of this group. Furthermore, future work should include molecular data on Rutelinae species to facilitate the systematic revision of this subfamily.

## 5. Conclusions

In this study, we sequenced the mitogenomes of four *Anomala* species (*A. aulax*, *A. vitalisi, Anomala* sp., and *A. xantholoma*) and combined them with those of Rutelinae for comparative analysis. The evolution of the mitogenomes of four *Anomala* species tended to be conservative, as indicated by the Ka/Ks values of 15 PCGs, which were all <1. In addition, we performed ML and BI analyses to construct phylogenetic relationships of Scarabaeidae based on different datasets (AA, PCG12 and PCG). The phylogenetic results supported the monophyly of Passalidae, Lucanidae, Geotrupidae, Trogidae, Hybosoridae, and Glaphyridae at the subfamily level, and indicated that the Melolonthinae subfamily is a dyadic group.

## Data Availability

The data that support the findings of this study will be available from GenBank at https://www.ncbi.nlm.nih.gov/, with accession number PQ139503-PQ139506 (*Anomala aulax* PQ139506, *Anomala* sp. PQ139505, *A. vitalisi* PQ139504, and *A. xantholoma* PQ139503).

## Supplementary Materials

The following supporting information can be downloaded at: https://www.mdpi.com/article/10.3390/genes17080949/s1, Figures S1–S5 show the phylogenetic trees inferred from different datasets. Table S1. Information about the specimens used in the study. Table S2. Information of samples for phylogenetic analyses. Figure S1, Molecular phylogeny of Scarabaeidae based on maximum likelihood analysis of 13 protein-coding genes using IQ-TREE version 1.6.12. Figure S2, Molecular phylogeny of Scarabaeidae based on Bayesian inference analysis of PCG12 (first and second codon positions of 13 PCGs) using MrBayes 3.2.7. Figure S3, Molecular phylogeny of Scarabaeidae based on maximum likelihood analysis of PCG12 (first and second codon positions of 13 PCGs) using IQ-TREE version 1.6.12. Figure S4, Molecular phylogeny of Scarabaeidae based on Bayesian inference analysis of AA (amino acid sequences of 13 PCGs) using MrBayes 3.2.7. Figure S5, Molecular phylogeny of Scarabaeidae based on maximum likelihood analysis of AA (amino acid sequences of 13 PCGs) using IQ-TREE version 1.6.12.
