# Supplementary material for: Comparative Mitogenomics of Four Anomala Species and a Phylogenetic Reassessment of Scarabaeidae
Source: Genes (Basel). 2026 Aug 13;17(8):949. doi: 10.3390/genes17080949 (PMC13512809; doi:10.3390/genes17080949)
Supplement: Supplementary file 1 [file genes-17-00949-s001.zip › Figures S1-S5.pdf]

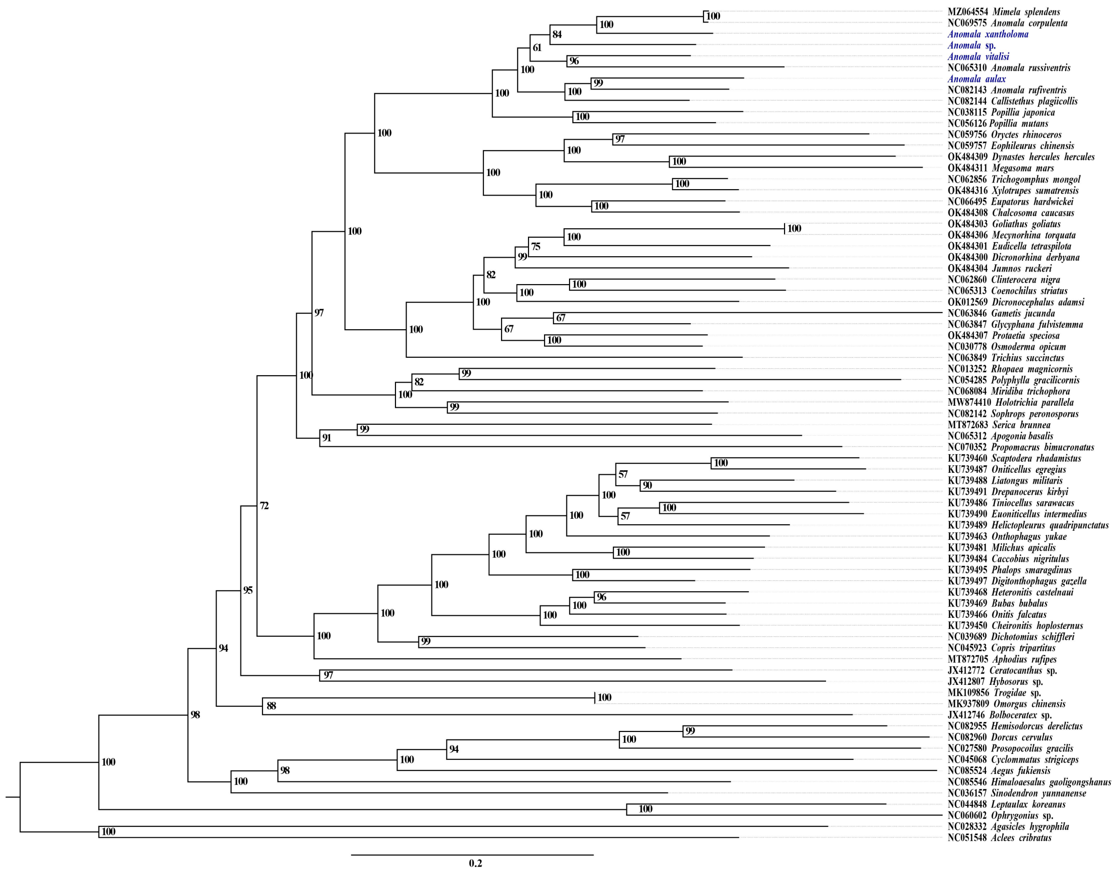

Figure S1 Molecular phylogeny of Scarabaeidae based on maximum likelihood analysis of 13 protein-coding genes using IQ-TREE version 1.6.12.

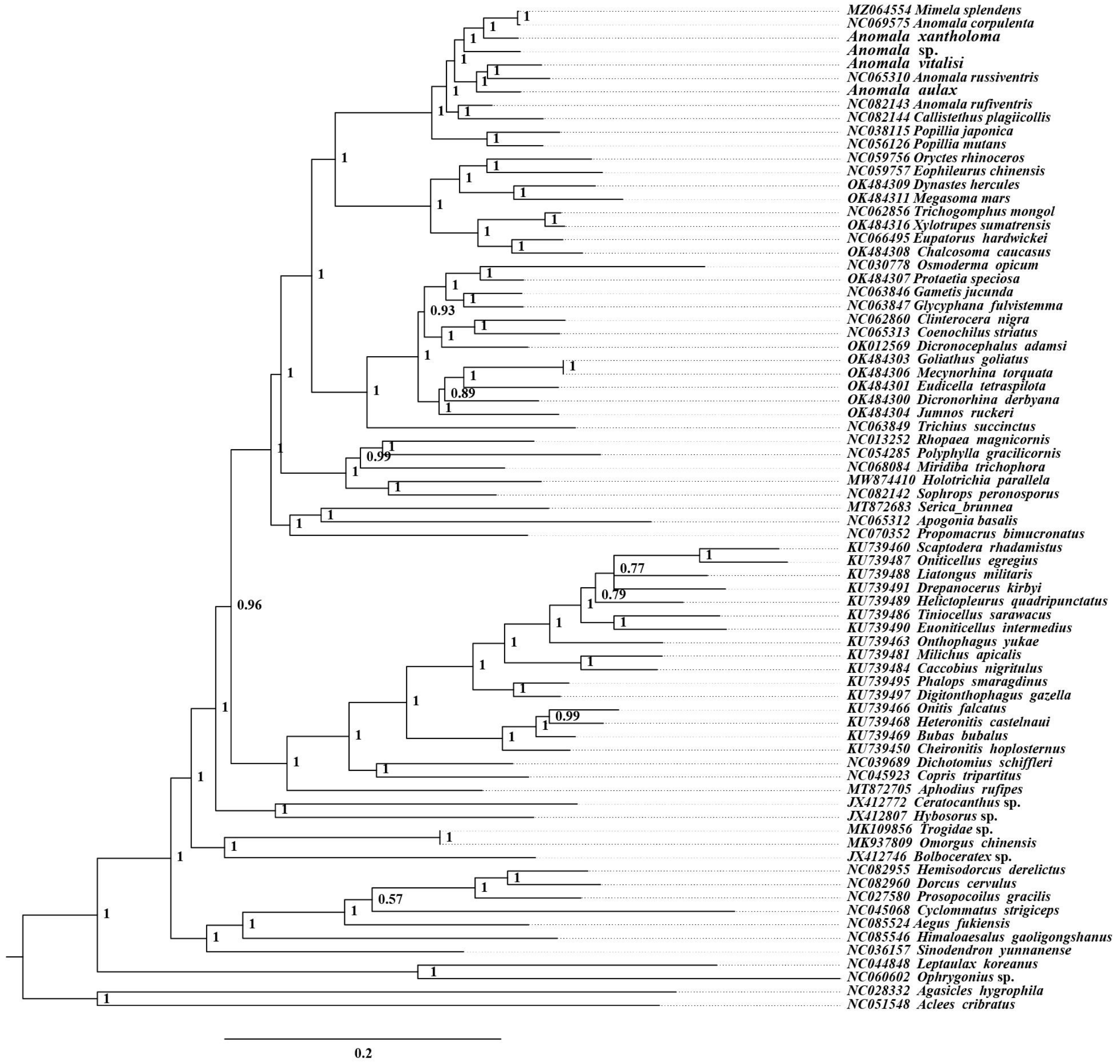

Figure S2 Molecular phylogeny of Scarabaeidae based on Bayesian inference analysis of PCG12 (first and second codon positions of 13 PCGs) using MrBayes 3.2.7.

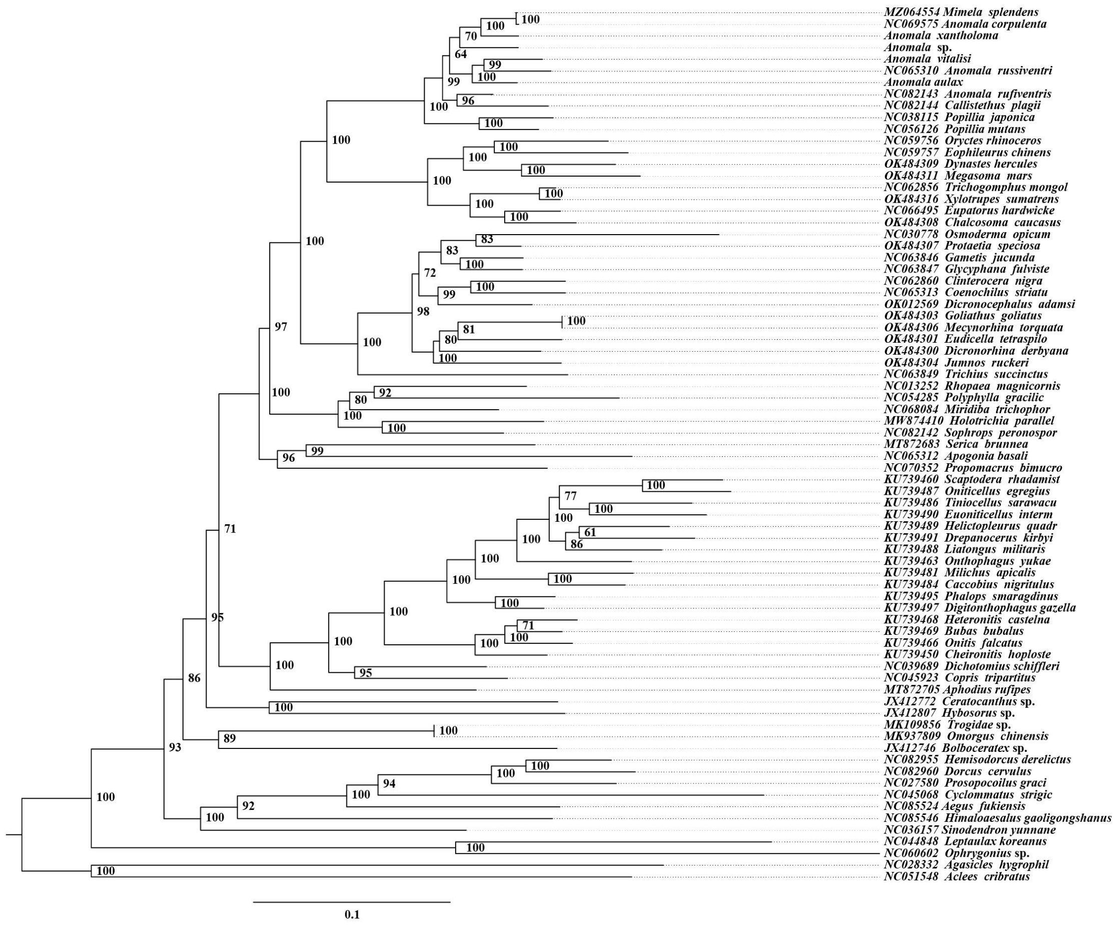

Figure S3 Molecular phylogeny of Scarabaeidae based on maximum likelihood analysis of PCG12 (first and second codon positions of 13 PCGs) using IQ-TREE version 1.6.12.

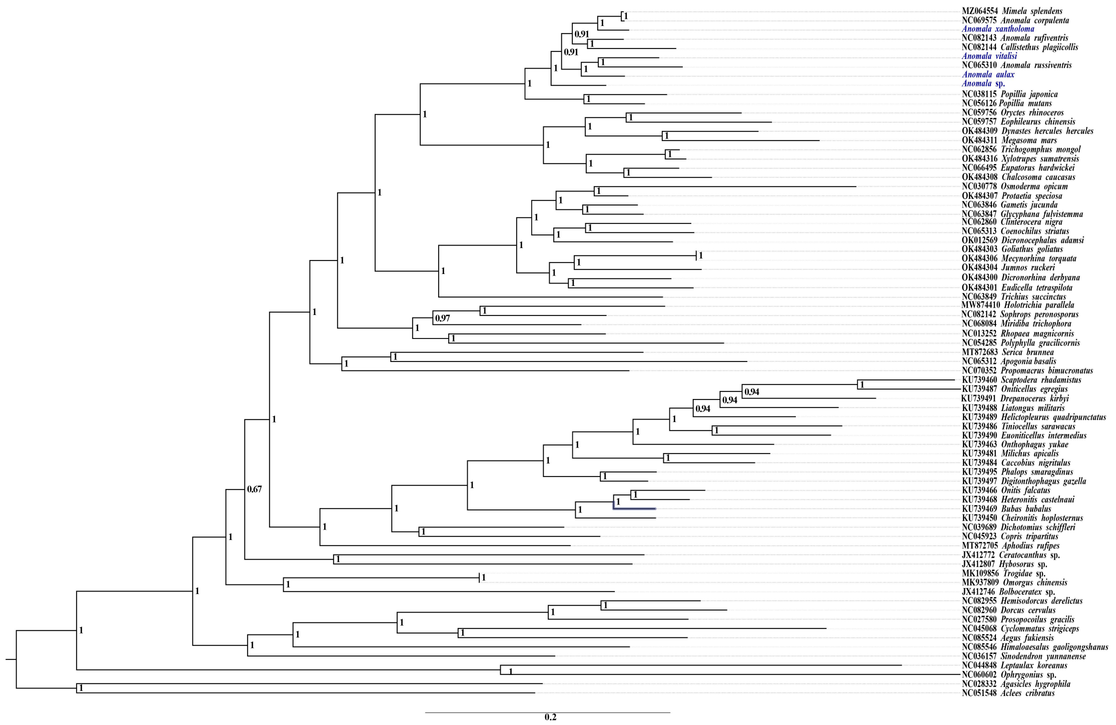

Figure S4 Molecular phylogeny of Scarabaeidae based on Bayesian inference analysis of AA (amino acid sequences of 13 PCGs) using MrBayes 3.2.7.

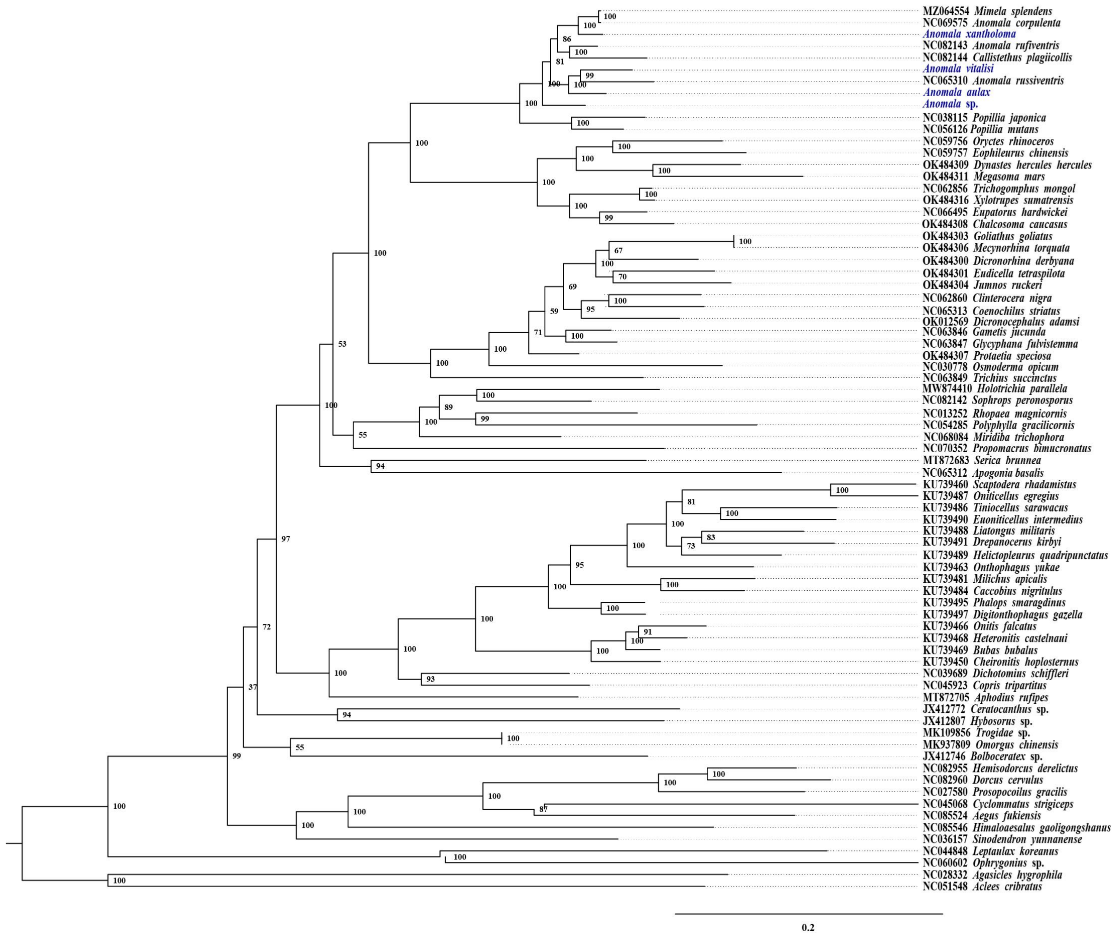

Figure S5 Molecular phylogeny of Scarabaeidae based on maximum likelihood analysis of AA (amino acid sequences of 13 PCGs) using IQ-TREE version 1.6.12.
