# Supplementary material for: Comparative Mitogenomics of Four Anomala Species and a Phylogenetic Reassessment of Scarabaeidae
Source: Genes (Basel). 2026 Aug 13;17(8):949. doi: 10.3390/genes17080949 (PMC13512809; doi:10.3390/genes17080949)
Supplement: Supplementary file 1 [file genes-17-00949-s001.zip › Table S1 Information about the specimens used in the study.pdf]

Table S1 Information about the specimens used in the study

Species	Species collection site	Collection time	Certificate specimen number
Anomala aulax	Honghe City, Yunnan Province, China	June 2022	GZ036
Anomala sp.	Tongren City, Guizhou Province	July 2020	GZ076
Anomala vitalisi	Zunyi City, Guizhou Province	August 2021	GZ093
Anomala xantholoma	Honghe City, Yunnan Province, China	June 2022	GZ039
