# Supplementary material for: Comparative Mitogenomics of Four Anomala Species and a Phylogenetic Reassessment of Scarabaeidae
Source: Genes (Basel). 2026 Aug 13;17(8):949. doi: 10.3390/genes17080949 (PMC13512809; doi:10.3390/genes17080949)
Supplement: Supplementary file 1 [file genes-17-00949-s001.zip › Table S2. Information of samples for phylogenetic analyses..pdf]

Table S2. Information of samples for phylogenetic analyses.

Family	Taxa	Species	Genebank	References
	Ceratocanthinae	*Ceratocanthus* sp.	JX412772	1
Geotrupidae	Geotrupidae	*Bolboceratex* sp.	JX412746	1
Glaphyridae		*Propomacrus bimucronatus*	NC070352	-
Hybosoridae		*Hybosorus* sp.	JX412807	1
Lucanidae	Aesalinae	*Himaloaesalus gaoligongshanus*	NC085546	2
	Lucaninae	*Aegus fukiensis*	NC085524	-
		Cyclommatus strigiceps	NC045068	-
		Dorcus cervulus	NC082960	-
		Hemisodorcus derelictus	NC082955	-
		Prosopocoilus gracilis	NC027580	3
	Syndesinae	*Sinodendron yunnanense*	NC036157	4
Passalidae		*Leptaulax koreanus*	NC044848	5
		Ophrygonius sp.	NC060602	-
Scarabaeidae	Aphodiinae	*Aphodius rufipes*	MT872705	-
	Cetoniinae	*Clinterocera nigra*	NC062860	6
		Coenochilus striatus	NC065313	7
		Dicronocephalus adamsi	OK012569	8
		Dicronorhina derbyana	OK484300	9
		Eudicella tetraspilota	OK484301	9
		Gametis jucunda	NC063846	-
		Glycyphana fulvistemma	NC063847	-
		Goliathus goliatus	OK484303	9
		Jumnos ruckeri	OK484304	9
		Mecynorhina torquata	OK484306	9
		Osmoderma opicum	NC030778	10
		Protaetia speciosa	OK484307	9
		Trichius succinctus	NC063849	-
	Dynastinae	*Chalcosoma caucasus*	OK484308	9
		Dynastes hercules	OK484309	9
		Eophileurus chinensis	NC059757	-
		Eupatorus hardwickei	NC066495	-
		Megasoma mars	OK484311	9
		Oryctes rhinoceros	NC059756	-
		Trichogomphus mongol	NC062856	-
		Xylotrupes sumatrensis	OK484316	9
	Melolonthinae	*Apogonia basalis*	NC065312	-

Family	Taxa	Species	Genebank	References
		Holotrichia parallela	MW874410	-
		Miridiba trichophora	NC068084	-
		Polyphylla gracilicornis	NC054285	-
		Rhopaea magnicornis	NC013252	11
		Sophrops peronosporus	NC082142	12
	Rutelinae	*Anomala corpulenta*	NC069575	13
		Anomala rufiventris	NC082143	12
		Callistethus plagiicollis	NC082144	12
		Anomala aulax		This study
		Anomala vitalisi		This study
		Anomala xantholoma		This study
		Anomala sp.		This study
		Anomala russiventris	NC065310	7
		Mimela splendens	MZ064554	-
		Popillia japonica	NC038115	14
		Popillia mutans	NC056126	15
	Scarabaeinae	*Copris tripartitus*	NC045923	16
		Euoniticellus intermedius	KU739490	17
		Helictopleurus quadripunctatus	KU739489	17
		Liatongus militaris	KU739488	17
		Oniticellus egregius	KU739487	17
		Tiniocellus sarawacus	KU739486	17
		Caccobius nigritulus	KU739484	17
		Digitonthophagus gazella	KU739497	17
		Milichus apicalis	KU739481	17
		Onthophagus yukae	KU739463	17
		Phalops smaragdinus	KU739495	17
		Scaptodera rhadamistus	KU739460	17
		Bubas bubalus	KU739469	17
		Cheironitis hoplosternus	KU739450	17
		Dichotomius schiffleri	NC039689	18
		Drepanocerus kirbyi	KU739491	17
		Heteronitis castelnaui	KU739468	17
		Onitis falcatus	KU739466	17
	Sericinae	*Serica brunnea*	MT872683	-
	Trogidae	*Trogidae* sp.	MK109856	-
		Omorgus chinensis	MK937809	-

Family	Taxa	Species	Genebank	References
Outgroup	Cucujiformia	*Agasicles hygrophila*	NC028332	19
		Aclees cribratus	NC051548	20

"-" represents unpublished

1. Timmermans M.J.; Barton C.; Haran J.; Ahrens D.; Culverwell C.L.; Ollikainen A.; Dodsworth S.; Foster P.G.; Bocak L.; Vogler A.P. Family-Level Sampling of Mitochondrial Genomes in Coleoptera: Compositional Heterogeneity and Phylogenetics. *Genome Biol Evol.* **2015**, 8, 161-75.

2. Jafir M.; Zhou L.; Chen Y.; Wan X. The first mitogenomic phylogenetic framework of Dorcus sensu lato (Coleoptera: Lucanidae), with an emphasis on generic taxonomy in Eastern Asia. *BMC Ecol Evol.* **2024**, 24(1): 66.

3. Wu Y.Y.; Cao Y.Y.; Fang J.; Wan X. The first complete mitochondrial genome of stag beetle from China, Prosopocoilus gracilis (Coleoptera, Lucanidae). *Mitochondrial DNA A DNA Mapp Seq Anal.* 2016, 27, 2633-4.

4. Lin Z.Q.; Song F.; Li T.; Wu Y.Y.; Wan X. New Mitogenomes of Two Chinese Stag Beetles (Coleoptera, Lucanidae) and Their Implications for Systematics. *J Insect Sci.* 2017, 17, 63.

5. Lee, S.G.; Choi, K.S.; Kim, C.J.; Jeon, H.B.; Kim, I.K. Complete mitochondrial genome of Leptaulax koreanus (Coleoptera: Passalidae), a Korean endemic bess beetle. *Mitochondrial DNA Part B.* **2018.** 4. 105-106.

6. Wu L.W.; Chen M.Y.; Li C.L. Phylogenetic position and morphological polymorphism of the chafer, Clinterocera nigra (Coleoptera: Scarabaeidae: Cetoniinae) from Taiwan. *Mitochondrial DNA B Resour.* **2022** 7, 1513-1515.

7. Li Y., Nie R.E., Lu Y., Lee S., Zhao Z., Wu L., Sun H., Bai M.. Seven new mitochondrial genomes of phytophagous scarab beetles (Coleoptera: Scarabaeidae) and phylogenetic implications. *Zootaxa* **2022**, 5138, 324-338.

8. Guo S.; Lin X.; Song N. Mitochondrial phylogenomics reveals deep relationships of scarab beetles (Coleoptera, Scarabaeidae). *PLoS ONE* **2022**, 17, e0278820.

9. Ayivi S.; Tong Y.; Storey K.B.; Yu D.N.; Zhang J.Y. The mitochondrial genomes of 18 new Pleurosticti (Coleoptera: Scarabaeidae) exhibit a novel trnQ-NCR-trnI-trnM gene rearrangement and clarify phylogenetic relationships of subfamilies within Scarabaeidae. *Insects*. **2021**, 12, 1025.

10. Kim M.J., Jeong S.Y., Jeong J.C., Kim S.S., Kim I. Complete mitochondrial genome of the endangered flower chafer *Osmoderma opicum* (Coleoptera: Scarabaeidae). *Mitochondrial DNA B.* **2016**,1, 148–149.

11. Cameron, Stephen & Sullivan, Jaron & Song, Hojun & Miller, Kelly & Whiting, Michael. (2009). A mitochondrial genome phylogeny of the Neuropterida (lace‐wings, alderflies and snakeflies) and their relationship to the other holometabolous insect orders. Zoologica Scripta. **2009**, 38. 575 - 590.

12. Long T.; Zhu W.; Yang L.; Long J.; Chang Z.; Chen X. First report of the complete mitochondrial genome of 3 beetles (Coleoptera: Scarabaeidae) harming Gastrodia elata (Asparagales: Orchidaceae). *Journal of insect science*. **2024**. 24, 12

13. Qu C.J.; Zhu Y.; Jiang C.; Qu M.J.; Wang X.Y.; Li X. Whole Mitochondrial Genome and Phylogeny Analysis of *Anomala corpulenta*. *Biotechnology Bulletin.* **2023**, 39, 263-273.

14. Yang W.; Zhang Y.; Feng S.; Liu L.; Li Z. The first complete mitochondrial genome of the Japanese beetle *Popillia japonica* (Coleoptera: Scarabaeidae) and its phylogenetic implications for the superfamily Scarabaeoidea. *International Journal of Biological Macromolecules.* **2018**, 118, 1406-1413.

15. Song, N.; Zhang, H. The mitochondrial genomes of *phytophagous* scarab beetles and systematic implications. *Journal of Insect Science*. **2018**, 18, 11.

16. Jeong J.S.; Kim M.J.; Kim I. The mitochondrial genome of the dung beetle, *Copris tripartitus*, with mitogenomic comparisons within Scarabaeidae (Coleoptera). *International journal of biological macromolecules.* **2020,** 144, 874-891.

17. Amorim I.C.; Melo A.S.; Cruz G.A.D.S; Wallau G.D.L.; de Moura R.C. *Dichotomius* (luederwaldtinia) schiffleri (coleoptera: scarabaeidae) mitochondrial genome and phylogenetic relationships within the superfamily scarabaeoidea. *Mitochondrial DNA B.* **2017**, 2, 887-888.

18. Breeschoten T.; Doorenweerd C.; Tarasov S.; Vogler A.P. Phylogenetics and biogeography of the dung beetle genus *Onthophagus* inferred from mitochondrial genomes. *Mol Phylogenet Evol.* **2016**, 105, 86–95.

19. Li N.; Wei J.N.; Jia D.; Li S.; Ma R.Y. The complete mitochondrial genome of the flea beetle *Agasicles hygrophila*. *Mitochondrial Dna Part A Dna Mapping Sequencing & Analysis*. **2016,** 27, 3670.

20. Wang B.X.; Xu Y.L.; Zhuo Z.H.; Xu X.L.; Liu J.; Qiu J.; Fang R.; Liu Y.K.; Zeng Z.; Xiao Q.G. The complete mitochondrial genome of the fig weevil, *Aclees cribratus* (coleoptera: curculionidae). *Mitochondrial DNA Part B*. **2020**, 5, 2599-2600.
